# Love of Neighbor During a Pandemic: Navigating the Competing Goods of Religious Gatherings and Physical Health

**DOI:** 10.1007/s10943-020-01031-6

**Published:** 2020-05-13

**Authors:** Tyler J. VanderWeele

**Affiliations:** Harvard T.H. Chan School of Public Health, Human Flourishing Program, Harvard University, 677 Huntington Avenue, Boston, MA 02115 USA

**Keywords:** Religion, Service attendance, Pandemic, Health, Love, Hope, Suffering

## Abstract

In light of the present pandemic, many religious communities have been asked to suspend their services and meetings. From the perspective of these communities, this comes at considerable cost to the spiritual good that these religious services bring about. Empirical evidence also indicates that the suspension of these services will have costs concerning physical and mental health as well. However, in the case of a pandemic, because it is an *infectious* disease that is the concern, love of neighbor arguably does entail the suspension of services for the sake of the preservation of life for others. Religious communities and individuals can, and have, found ways to partially offset the losses from not being able to meet. These have included increased personal and family prayer and devotion, video-streaming of services, and online prayer and discussion meetings. While none of these fully compensates for the loss of in-person meetings, the sacrifice entailed may itself be seen as a means to greater love of God and love of neighbor.

## Commentary

Many religious communities currently find themselves in a difficult position: their principal activity of meeting together in religious services is being severely limited by the present pandemic. Services have, in many places around the world, been suspended so as prevent the spread of coronavirus (COVID-19). To some secular commentators, this closing of churches, synagogues, and mosques seems like an obvious conclusion—surely, they argue, these “inessential services” can be postponed, just as sports matches, until the crisis is under control. For believers, the situation is more complicated. These religious services are not just social gatherings, like a weekly card game with friends. Rather the religious services constitute a critical means to what is considered the most important ultimate end of communion with God. Believers might well argue, and many have, that the spiritual goods that they seek are in fact more important than physical health. While the arguments considered below concern a distinctively Christian perspective, analogous issues arguably arise in other religious traditions as well. Within the Christian tradition, for example, a case might be mounted on Biblical grounds. In the words of Jesus in the Gospel of Saint Mark, “For what does it profit a man to gain the whole world and forfeit his soul?”, or in the first letter to Timothy ascribed to Saint Paul, “while bodily training is of some value, godliness is of value in every way, as it holds promise for the present life and also for the life to come” (1 Tim. 4:8). Surely then, some argue, is it not worth risking the good of physical health for something of far greater importance?

The difficulty with such arguments in the case of the present pandemic is that it is not only one’s own physical health that one puts at risk, but potentially also that of one’s community, and even one’s nation and the world. Because this is not just a disease, but an *infectious* disease, there is more at stake than one’s own health. Unfortunately, the types of, often large, meetings that religious communities typically hold are precisely the types of events wherein infection can most easily spread. And it is clear that the present virus is highly contagious. It is contagious without symptoms. It is contagious not only through physical touch, but also can be transmitted by breath through the air. And the spread to many at religious services will then be magnified several times over as each participant goes on to see others, purchases groceries, returns home, and so forth. By continuing to meet, religious communities put not only themselves at risk but the entire community.

The relevant theological and Biblical principle governing the decisions of religious communities in this matter is arguably that of love of neighbor. In this case, love of neighbor arguably entails the present foregoing, for some time, of communal religious gatherings. Jesus’ response, when being questioned as to the greatest commandment, was “‘You shall love the Lord your God with all your heart and with all your soul and with all your mind and with all your strength.’ The second is this: ‘You shall love your neighbor as yourself.’” (Mark 12:30–31). Sometimes love of God and love of neighbor have been pitted against one another in these discussions of the appropriate response of religious communities during the pandemic, with love of God being seen as the greater commandment, thereby justifying the continuing of religious services. In the Gospel of Saint Luke, the two are in fact presented as a single commandment: “You shall love the Lord your God with all your heart and with all your soul and with all your strength and with all your mind, and your neighbor as yourself” (Luke 10:27) and even in the Gospels of Saint Mark and Saint Matthew, wherein they are presented as two commandments, they are still both given in response to the question concerning *the* greatest commandment. They are deeply intertwined. In the understanding of Saint Thomas Aquinas, charity, love of God, entails love of neighbor (Aquinas 1274/1948). Neglect of love of neighbor is also in fact neglect of love of God. Such a view is likewise found in the first Epistle of John: “He who does not love his brother whom he has seen cannot love God whom he has not seen” (1 John 4:20).

Human life is often seen as the highest good of this world and is defended at great cost. In the case of the present pandemic, the risks to human life are high. This is not merely a matter of someone feeling a bit unwell for a time. For many, and especially for the elderly, it can be a matter of life and death. One would not run over someone in one’s car to make it to church. Likewise, one ought not, when the pandemic is near its peak, risk the lives of many in order to meet. While individual mortality risk with coronavirus may not be especially high, because this is again an *infectious* disease, the risk of its spread to many, and thus killing some (especially the elderly and vulnerable) is considerable. Love of neighbor entails concern for the life of one’s neighbor. Thus, at present, love arguably, and sadly, entails the foregoing of meetings. This is not the foregoing of spiritual life, and there are many responses that individuals and religious communities can pursue to help preserve important spiritual goods. For many, however, the absence of communal religious services will constitute a time of trial. It is a trial worth confronting. Jesus himself spent forty days away from community, being tested. Such trials and testing can ultimately result in a strengthening of one’s faith, but such trials need to be confronted in ways that best facilitate the spiritual life, even in the midst of these difficulties.

What then should the response of religious communities be? First, there should certainly be an acknowledgement of real loss. There is loss of spiritual good that comes from religious services; there is loss of communal life; there is arguably also a loss of physical and mental health and well-being associated with not meeting. Numerous recent rigorous studies have suggested relatively strong associations between participation in religious services with greater longevity, less depression, less suicide, greater meaning, greater generosity, greater civic engagement, and numerous other outcomes (Koenig et al. 2012; Idler 2014; VanderWeele 2017). Of all the various aspects of religion and spirituality, it appears moreover to be religious service attendance that is most predictive of these (Musick et al. 2004; VanderWeele et al. 2017). And it is of course such services that are under threat. There are real losses here and these must be acknowledged. There is sacrifice involved in the giving up of these various goods to help attain other goods—namely, the preservation of life. Moreover, it is not only goods of this life that are under threat by being unable to meet, but also spiritual goods as well.

There are, however, ways that believers can help counter the full consequences of the loss of not being able to meet. Periods of mandated social distancing and even lockdown can be opportunities to increase personal devotions and prayer. They can be opportunities for new family religious ritual and practice. Many communities are making services and masses available online through live-streaming. Some have begun online studies of Scriptures and prayer meetings. Others have instituted “drive-through” prayer, and even confession. Spiritual direction might similarly take place through modern video-conferencing options.

Even in this present time of crisis it may also still be possible to administer the sacraments in very small gatherings. A baptism with only the immediate family present poses risk not very different from a trip to the grocery store. A gathering of four is very different than a gathering of four hundred. Weddings are often joyful communal celebrations, but if there are compelling reasons not to delay a marriage, a small private wedding with only the bride and groom and perhaps a couple of additional family members might likewise be considered reasonable.

However, just because there are some ways to continue to encourage and promote spiritual life at present does not mean that everything can be fully compensated. Perhaps most notably, participation in the Eucharist cannot be easily carried out under the present constraints. This is a real loss. There is no fully adequate substitute here. The Catechism of the Catholic Church acknowledges the possibility of this very real loss: “If…for other grave cause participation in the celebration of the Eucharist is impossible, it is specially recommended that the faithful…engage in prayer for an appropriate amount of time personally or in a family or, as occasion offers, in groups of families” (Catechism of the Catholic Church 2000). This will not fully compensate, but it can provide additional motivation to continue to pursue, in more substantial ways, personal and family prayer and devotion.

The loss of important spiritual goods can then be partially, though not fully, offset. Likewise, the loss of other goods that accompany religious services can be partially offset. With regard to the beneficial effects of religious participation on physical, mental, and social health and well-being, there are ways in which the practices that are still available at present might still help bring some of this about. The evidence with regard to the mechanisms by which religious services affect physical health and longevity suggest that there may be many important pathways (Li et al. 2016; Morton et al. 2017; Kim and VanderWeele 2019). One of these is social support and connection. And even at the present time, while more difficult, social connection is still possible. One can make an effort to stay in touch with friends and family members, and have meaningful conversations, by phone or using online video-conferencing programs. One can additionally invest in a spouse or child with the time that may now be available. One can connect online with members of one’s religious community. All of this is still within reach. Another important mechanism appears to be religious services promoting healthier behaviors, perhaps especially lower smoking rates (Strawbridge et al. 2001; Li et al. 2016). One can try as best as possible to pursue healthy lifestyles, recognizing the body as a gift from God and a temple of the Spirit. Yet another mechanism may be increased meaning and purpose. The present time can be used as one of reflection and re-orientation to what is most important in life, a renewal of commitment to love of God and love of neighbor as central. Another mechanism may be the encouraging of forgiveness; one can use this time to reflect on which relationships may be in need of forgiveness and reconciliation and one can pursue this. Helpful downloadable tools are now available online to assist in this (Worthington 2020; VanderWeele 2018). And yet another mechanism might be an increased sense of hope (Everson et al. 1996; Long et al. 2020), a conviction that in spite of the present difficulties, we may still find good in our present circumstances, we may grow from them, and we may await and seek more ultimate final goods to be found in God.

Each of these pathways by which religious services may promote health and well-being can be pursued even in the present time of crisis. There will again be no full substitutes. Social support and connection will be partially limited by the lack of ability to meet in person. The communal reinforcement of healthy behaviors arising from in-person time with others may not be fully operative. The meaning derived from being at a religious service can perhaps only be partially fulfilled by the online options. Thus, while some of the losses can be offset, others cannot. That nothing can fully take the place of the communal face-to-face gatherings of religious communities should likewise be kept in mind once the present crisis is over. There may be temptation for some to continue online-only. Some of the goods and benefits of religious service attendance from such online venues may be retained, but others not. After the present crisis, everyone should make effort to fully restore the vibrant life of their churches and religious communities.

Religious communities should also prepare for the ending of the most strict lockdown measures. There may be a time when full lockdown is over, but certain social distancing measures are still in place. For example, even after full lockdown measures are relaxed, it is possible that large meetings will still be prohibited. There may be a period during which, in certain cities or regions, only gatherings of 20–50 individuals are allowed. These measures may again be necessary to ensure that a second wave of the pandemic does not become too severe. Churches and other religious communities can prepare for these possibilities. It might be possible to hold multiple services or masses each day, including weekdays, so as to reduce the total number present at any given service. Particular care will likely be needed on Holy Days or days of the week of regular religious gatherings. Churches will have to consider how to appropriately handle Sunday mornings. Synagogues will need to wrestle with similar issues on Friday evenings and Saturday mornings. Rotating schedules or online sign-ups may help. In churches, care will have to be given with regard to the mode of administration of the Eucharist and of extending a sign of peace to others. It will be important to emphasize to parishioners or members the importance of continuing with whatever guidelines the religious community puts in place so as to avoid risking possible closure. In these cases, love of neighbor of course extends to members of one’s own religious community.

Religious communities can also advocate for better data on which policy-makers can base informed decisions. There are tremendous social, economic, and spiritual consequences to the present state of lockdown in many places. Some of these negative social, spiritual, and even health-related costs come from the suspension of religious services. We need better data to know how substantial the mortality risk truly is, how many have actually been infected, and when it is safe to begin to relax social distancing measures. The amount of uncertainty around the inputs being used in the modeling and in the policy decisions is substantial. If systems were put in place to allow representative testing, this could be radically altered (Pearce et al. 2020), and decision-making could be improved and the social, economic, and spiritual costs to society lessened. We should have such systems in place. The costs to society, to the economy, to individuals, and to religious communities are too high to not be better informed. Religious communities, and others, can point to these costs to advocate for better systems for gathering much needed information.

Until that time when religious communities can again meet together to hold religious services in-person, face-to-face, we must however acknowledge that there will be real losses without these meetings. There will be losses to the pursuit of spiritual life and the means to attain it; there will be losses to social connectedness; there will be losses to health. These losses are endured for the sake of love—to preserve the lives of others. It is a giving up of oneself on behalf of another, even though those other persons may, or may not, be directly known to us. These losses will entail suffering, but suffering too can bring with it new growth, a greater hope, a refined set of commitments and purposes, and an empathy oriented toward sharing in the sufferings of others. Our present set of circumstances entails suffering. Love requires accepting this suffering, helping others in the midst of it, and being transformed by it. There are things that can be done to pursue the spiritual life even in the midst of the loss of large religious gatherings. Love of God and love of neighbor allows us to find ways forward toward more ultimate ends.
